# Synergistic effect of smoking on age-related hearing loss in patients with diabetes

**DOI:** 10.1038/s41598-020-75880-2

**Published:** 2020-11-03

**Authors:** Seong Hoon Bae, Sang Hyun Kwak, Jae Young Choi, Jinsei Jung

**Affiliations:** 1 Department of Otorhinolaryngology, Yonsei University College of Medicine, 50-1 Yonsei-ro, Seodaemun-gu, Seoul, 03722 Republic of Korea; 2 Graduate School of Medical Science, Brain Korea 21 Project, Yonsei University College of Medicine, Seoul, Republic of Korea; 3 Department of Otorhinolaryngology, St. Vincent Hospital, College of Medicine, The Catholic University of Korea, Suwon, Republic of Korea

**Keywords:** Auditory system, Geriatrics, Risk factors, Neurodegenerative diseases, Diabetes

## Abstract

This study investigated the synergistic effects of risk factors on age-related hearing loss (ARHL) using nationwide cross-sectional data of 33,552 individuals from the 2010‒2013 Korea National Health and Nutrition Examination Survey. Patients with ARHL were selected based on their pure-tone audiometry results. Previously reported risk factors for ARHL were analyzed using logistic regression and propensity score-matching, and synergistic effects between risk factors were analyzed using propensity score-matching. Of the 12,570 individuals aged 40–79 years, 2002 (15.9%) met the criteria for ARHL. Male sex, exposure to occupational noise, and diabetes showed a significant relationship with ARHL (*p* < 0.05) in both the logistic regression and propensity score-matching analyses. Smoking and diabetes showed the strongest significant synergistic effect on ARHL (odds ratio [OR] 1.963, 95% confidence interval [CI] 1.285‒2.998; *p* = 0.002). In the subgroup analysis based on smoking status, current smokers with diabetes had a significant relationship with ARHL (OR 1.883, CI 1.191‒2.975; *p* = 0.009), whereas ex-smokers with diabetes did not (OR 1.250; CI 0.880‒1.775; *p* = 0.246). This implies that current smokers with diabetes may benefit from the cessation of smoking. In conclusion, patients with diabetes should strictly avoid or cease smoking to prevent the progression of ARHL.

## Introduction

Age-related hearing loss (ARHL) is the most common cause of sensory hearing impairment in older adults. It affects tens of millions of people worldwide, and an estimated one-third of people over the age of 65 years currently have hearing loss^1,2^. Several recent studies have suggested an association between hearing loss and age-related diseases, such as cognitive impairment, depression, increased risk of falling, etc.^3^, which raises the importance of hearing loss as a public health problem. Furthermore, the number of patients with ARHL continues to increase markedly. In 2020, an estimated 56% of individuals aged 70 years or older have ARHL, and this percentage is expected to increase to 67% by 2060^1^. Symptoms of ARHL develop at approximately 50 years of age and gradually worsen, without recovery.

Several previous cohort and cross-sectional studies have suggested risk factors for ARHL that have been categorized as genetic, environmental, and medical^4^. Genetic risk factors include sex and genetic mutations. A previous prospective study has shown that men are at a higher risk of developing ARHL than women^5^. Additionally, several reports have elucidated the association between genetic mutations and ARHL, although these cannot adequately explain the high incidence of ARHL. Occupational noise exposure is an environmental risk factor, and susceptibility to noise can differ among individuals. The association between noise exposure and ARHL is generally accepted, and the use of ear protection devices in noisy environments is recommended^6,7^. Smoking has also been suggested to be an environmental risk factor for ARHL^4,8^. Furthermore, various medical risk factors have been studied and suggested, such as stroke^9^, cardiovascular disease (CVD)^9,10^, hypertension (HTN)^11^, diabetes mellitus^12^, obesity^13^, and dyslipidemia^14,15^. However, the effects of some of these proposed risk factors remain controversial, perhaps due to the complex etiologies of ARHL and the small sample sizes of previous studies^16,17^. Furthermore, the synergistic effects of these risk factors have not been studied in-depth, which may be crucial in explaining the pathophysiology of ARHL in humans.

This study utilized the data of 33,552 individuals included in the nationwide annual report of the 2010‒2013 Korea National Health and Nutrition Examination Survey (KNHANES)^18^ to identify the synergistic interrelationship between the risk factors for ARHL. Using this population-based cross-sectional data, we analyzed the odds ratio (OR) for each combination of modifiable risk factors for ARHL, in order to substantiate the deleterious combinatorial effects of these risk factors. The propensity score-matching (PSM) model was preferably chosen for this analysis, as it substantially minimizes the collinearity of variables, which might be underestimated in the logistic regression model, and can be ideally applied to measure the accurately quantified OR of the combined risk factors for ARHL.

## Results

### Demographic information and evaluation of risk factors

The KNHANES is an annual nationwide population-based cross-sectional health examination and survey. In the 2010‒2013 survey, 33,552 individuals were investigated without a serial follow-up. Among them, a total of 12,570 individuals were included in this study; of these, 2002 (15.9%) individuals met the criteria for inclusion in the ARHL group. The mean participant age was 57.21 ± 10.86 years, and 43.6% of the participants were males.

Ten candidate variables that have previously been suggested as risk factors for ARHL were investigated (Supplementary Table S1 online). Environmental risk factors such as smoking history and occupational noise exposure history were investigated; 2296 (18.3%) individuals were current smokers, and 1755 (14.0%) individuals had experienced occupational noise exposure for > 3 months. Medical risk factors such as HTN, diabetes, dyslipidemia, stroke, CVD, and obesity were also investigated.

### Common risk factors for ARHL in logistic regression and propensity score-matching

We included the 10 proposed risk factors as independent variables and ARHL as a dependent variable in the regression model. The six variables (age, occupational noise exposure, stroke, male sex, current smoking, and dyslipidemia) included in the final regression model showed 84.8% accuracy of classification and a Nagelkerke’s R^2^ of 0.306 (i.e., 30.6% of events could be explained by this model) (Table 1). The *p* value, according to the Hosmer‒Lemeshow test, was 0.596, which indicated that this regression model was valid. In the regression model, occupational noise exposure showed the highest correlation with ARHL. The well-known cumulative effect of age on noise-induced hearing loss was consistent with this result^19^. Among the included risk factors, noise exposure (Exp [B] 1.722, CI 1.485‒1.998), male sex (Exp [B] 1.419, CI 1.262‒1.595), current smoking (Exp [B] 1.231, CI 1.057‒1.434), and diabetes (Exp [B] 1.168, CI 1.009‒1.351) showed a significant positive correlation with ARHL. Additionally, dyslipidemia (Exp [B] 0.808, CI 0.701‒0.931) showed a significant negative correlation with ARHL, although this result should be interpreted while taking the vascular pathology of dyslipidemia and its relationship with other diseases into consideration.

**Table 1:** Risk factors for age-related hearing loss in the logistic regression analysis model.

Significant variables	Exp (B)	95% CI	*P* value
Age	1.139	1.132–1.146	< 0.001
Occupational noise exposure	1.722	1.485–1.998	< 0.001
Male sex	1.419	1.262–1.595	< 0.001
Dyslipidemia	0.808	0.701–0.931	0.003
Current smoker	1.231	1.057–1.434	0.007
Diabetes	1.168	1.009–1.351	0.038

Accuracy of classification: 84.8%, Nagelkerke’s R^2^: 0.306, Hosmer‒Lemeshow test (*p* value): 0.596. CI, confidence interval.

In the PSM analysis, male sex, HTN, diabetes mellitus, and noise exposure showed significantly increased ORs (Table 2). Among these, noise exposure history had the highest OR (OR 1.779, CI 1.461‒2.167), which was consistent with the results of the logistic regression analysis. HTN (OR 1.157, CI 1.004‒1.333) showed a significantly increased OR in the PSM but not in the logistic regression analysis. The ARHL risk factors common to both statistic models were male sex, noise exposure, and diabetes.

**Table 2:** Odds ratios of age-related hearing loss for each risk factor in the propensity-score-matched groups.

Risk factor*	Pairs	OR	95% CI	*p*-value
Current smoke	1945	1.171	0.979–1.401	0.091
Diabetes*	1129	1.285	1.058–1.562	0.013
Dyslipidemia	1598	0.902	0.752–1.082	0.286
HTN*	2407	1.157	1.004–1.333	0.047
Stroke	242	1.080	0.735–1.588	0.768
CVD	107	0.709	0.382–1.314	0.348
Sex*	3178	1.434	1.252–1.642	< 0.001
Obese	3634	1.064	0.932–1.216	0.377
Noise*	1595	1.779	1.461–2.167	< 0.001

*CVD* cardiovascular disease; *CI* confidence interval; *HTN* hypertension; *OR* odds ratio.

### Synergistic effects of risk factors on ARHL

Next, we investigated the synergistic effects of the modifiable risk factors. Modifiable risk factors were defined as chronic diseases of lifestyle (HTN, diabetes, dyslipidemia, and obesity) and current smoking. Individuals who had both these targeted risk factors were compared to those who had a single or no targeted risk factor. Ten combinations of the five modifiable risk factors were investigated by PSM (Table 3). Notably, the combination of diabetes and current smoking was associated with the most significant increase in the prevalence of ARHL (OR 1.963, 95% CI 1.285–2.998; *p* = 0.002), after completely controlling for the other risk factors. The combination of diabetes and HTN was also statistically significant, but the OR (OR 1.388, 95% CI 1.100 − 1.750; *p* = 0.007) was smaller than that for the combination of diabetes and smoking. Other combinations showed no significant difference in terms of the prevalence of ARHL according to the presence of risk factors.

**Table 3:** Odds ratios of age-related hearing loss for each combination of two risk factors in the propensity score-matched groups.

Risk factor*	Pairs	OR	95% CI	*P* value
Diabetes + HTN^#^	723	1.388	1.100 –1.750	0.007
Diabetes + dyslipidemia	472	1.012	0.745–1.375	1.000
Diabetes + obesity	550	1.088	0.818–1.446	0.612
Diabetes + current smoker^#^	265	1.963	1.285–2.998	0.002
HTN + dyslipidemia	1042	0.977	0.791–1.207	0.871
HTN + current smoker	470	1.217	0.895–1.656	0.240
HTN + obesity	1571	1.105	0.931–1.311	0.274
Dyslipidemia + current smoker	241	1.029	0.644–1.644	1.000
Dyslipidemia + obesity	793	0.934	0.723–1.207	0.648
Current smoking + obesity	705	1.271	0.904–1.786	0.195

*Compared to the group with one or no risk factors; *CI* confidence interval; *HTN* hypertension; *OR* odds ratio; ^#^*p* value < 0.05.

**Figure 1:**
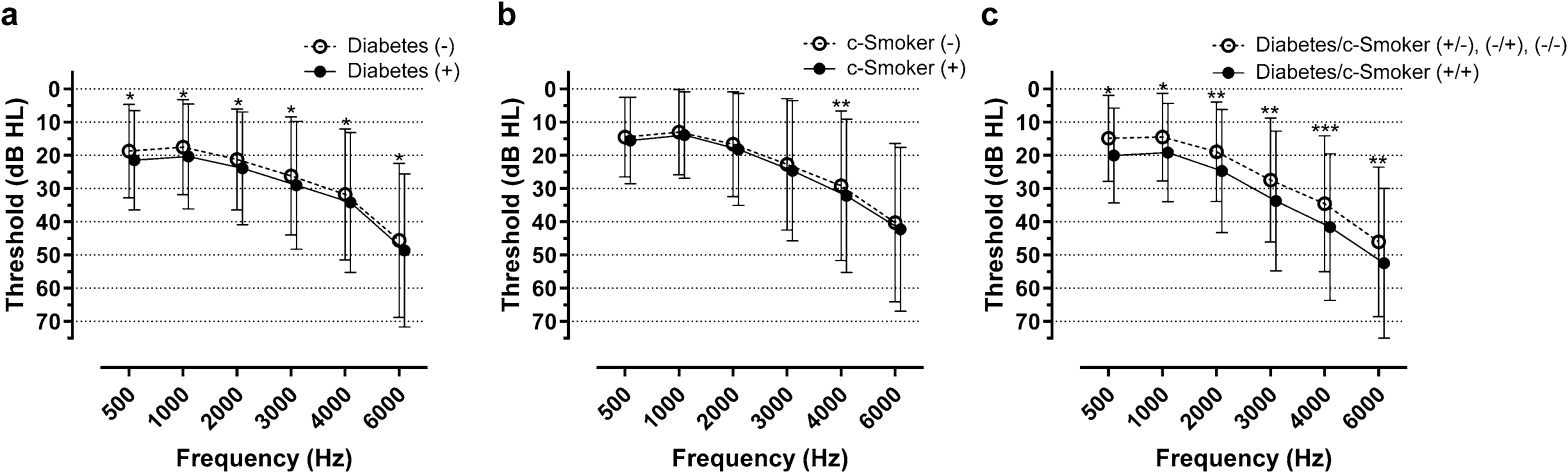
Pure-tone audiograms of propensity score-matched individuals in the analysis of the current smokers and never-smoked subgroups. Among 12,570 enrolled patients, 2296 current smokers and 7414 never-smoked individuals were included in the subgroup analysis. Propensity score-matching (PSM) was performed for age, male sex, hypertension, dyslipidemia, stroke, cardiovascular diseases, occupational noise exposure, and obesity as risk factors. In the propensity score-matched subjects, pure-tone thresholds were compared according to (**a**) the presence of diabetes (PSM pairs = 794), (**b**) current smoking status (PSM pairs = 950; denoted as c-Smoker in the figure), and (**c**) the combination of diabetes and current smoking status (PSM pairs = 230). c-Smoker, current smoker; * *p* < 0.05, ** *p* < 0.005, *** *p* < 0.001; paired *t*-test. The error bar indicates standard deviation.

Next, we performed a subgroup analysis of the prevalence of ARHL according to smoking status. The OR of ARHL did not differ between the ex-smokers and the never-smoked group (OR 1.008, 95% CI 0.789–1.287; *p* = 1.000) (Supplementary Table S2 online). Additionally, there was no synergistic effect between ex-smoking status and diabetes in terms of the OR of ARHL (OR 1.250; 95% CI, 0.880–1.775, *p* = 0.246) (Table 4). However, in the analysis of the current smokers and the never-smoked subgroups, a synergistic effect on the odds of developing ARHL was observed between current smoking status and diabetes (OR 1.883, 95% CI 1.191‒2.975; *p* = 0.009) (Table 4). The combined effect of current smoking status and diabetes on the hearing function is clearly presented in Fig. 1. Unlike current smoking status, the ex-smoking status did not increase the OR of ARHL in patients with diabetes.

**Table 4:** Subgroup analyses of odds ratios of age-related hearing loss according to smoking status in the propensity-score-matched groups.

Subgroup	Risk factor	Pairs	OR	95% CI	*p* value
Ex- and never-smoker	Ex-smoking	962	1.008	0.789 –1.287	1.000
	Ex-smoking + diabetes*	326	1.250	0.880–1.775	0.246
Current and never-smoker	Current smoking	950	1.216	0.941–1.572	0.151
	Current smoking + diabetes*^#^	230	1.883	1.191–2.975	0.009

*Compared to the group with one or no risk factors; *CI* confidence interval; *OR* odds ratio; ^#^*p* value < 0.05b.

## Discussion

In this study, we performed a PSM analysis and observed that the combination of smoking history and diabetes contributed most significantly to ARHL. Moreover, we observed that diabetic ex-smokers showed a reduced OR compared to current diabetic smokers. *In the PSM analysis, the OR of the combination of diabetes and current smoking (1.963) was higher than the ORs of current smoking (1.171), diabetes (1.285), and HTN (1.157) alone as well as the combination of diabetes and HTN (1.388).*Considering that both diabetes and smoking may affect cardiovascular circulation, the progression of ARHL may be attributed to previously suggested risk factors, such as HTN, dyslipidemia, stroke, obesity, and CVD^9,14,20^.

ARHL is a slowly progressive disease that develops over decades and has an extremely complex etiology. Although a definite causative gene contributing to the development of ARHL in the population is unknown, numerous genes with common polymorphisms or rare variants have been suggested as a cause for ARHL in some patients; for instance, *KCNQ4* is a candidate gene associated with hearing loss that progresses with age^16,21–29^. However, the genetic information of the subjects was not available in this study. The analysis of genetic polymorphisms associated with ARHL would be more helpful in substantiating the causative relations of the risk factors for ARHL in the future. Further, several environmental contributors to ARHL have been suggested, including ototoxic medication, alcohol intake, tobacco use, and head trauma^17^. Because ARHL has complex etiologies, the effects of some risk factors, such as diabetes and dyslipidemia, remain controversial^4,14,30^. In this study, dyslipidemia showed a negative correlation and no correlation with ARHL in the logistic regression and PSM analysis, respectively. Interestingly, dyslipidemia seems to have a lower association with ARHL, speculation of which has been supported by recent studies^31,32^. We also observed a discrepancy in the effects of some risk factors (HTN, smoking) on ARHL between logistic regression and PSM analyses. Additionally, other medical conditions, such as CVD, stroke, obesity, and dyslipidemia, did not contribute to the difference in the OR for ARHL in logistic regression and PSM analyses, which was inconsistent with the findings of previous literature^9–12^. This may indicate that several medical conditions have mutual effects, and no single effect from each risk factor can explain the pathology of ARHL. In this regard, we consider that multifactorial analyses may be more helpful in explaining the pathophysiology of ARHL. Considering that several risk factors, such as HTN, diabetes, CVD, dyslipidemia, obesity, and smoking, are associated with vascular disease and are interrelated, controlling for confounding factors is important when evaluating a single risk factor for ARHL^9,14,20^.

Meanwhile, the logistic regression analysis revealed that older age, noise exposure, male sex, current smoking, and diabetes were significantly associated with ARHL, whereas the PSM revealed that noise exposure, male sex, diabetes, and HTN significantly increase the OR of ARHL. Because the PSM and logistic regression analyses are complementary, it is reasonable to adopt the results from both methods. In particular, we used PSM analysis to confirm the hierarchical association of the known risk factors for ARHL without the interactions between covariates. PSM analysis was also preferably chosen for determining the synergistic effects of each risk factor, since PSM can substantially minimize the collinearity of variables, thereby accurately predicting the synergistic effects. Additionally, PSM can convincingly define an association hierarchy among varying combinations by perfectly matching the other co-variables between groups. Although the interaction regression model using the cross-product variable can be applied to evaluate the interactions of covariates, it is less appropriate in this study because too many medical conditions should be hypothesized as interacting conditions.

Smoking is a modifiable risk factor in preventing the progression of ARHL in patients with diabetes. In particular, we observed that ex-smoking status did not have a significant effect on the OR for ARHL in diabetic patients, indicating that ARHL may be alleviated if a patient quit smoking. The synergistic mechanism of smoking and diabetes in ARHL remains unclear, and the duration of smoking cessation that is required to obtain a reduced OR of ARHL is not known. In general, smoking and diabetes have synergistic harmful effects on blood circulation; smoking increases the progression of nephropathy and the risk of CVD in patients with diabetes^33–35^. It has also been reported that C-reactive protein, a systemic inflammation marker, is increased in current smokers compared to ex-smokers and those who have never smoked^36,37^. Moreover, a recent study has suggested that inflammatory food consumption is closely related to the incidence of ARHL^38^. Given that chronic inflammation increases the risk of cardiovascular events, as supported by the results of the JUPITER trial, smoking and diabetes may cause vascular pathologies, such as atherosclerosis^39^. This synergism may eventually destroy the vascular supply to the auditory end organ, the cochlea, and cause progressive hearing deterioration. Another possible mechanism may be direct cellular damage to the sensory hair cells in the cochlea or auditory neural pathway. In a mouse model, a high glucose level affects auditory midbrain function, loss of spiral ganglion cells, decreased cochlear blood flow, and increased susceptibility to noise exposure^40,41^. Smoking also increases susceptibility to noise exposure and decreases spiral ganglion cells due to increased oxidative stress in the mouse model^42,43^. According to the data in our study, cochlear damage due to chronic smoking may be reversed through cessation of smoking. Therefore, strict and timely control of smoking is advised, particularly in patients with diabetes.

A limitation of this study is that it did not provide specific information on the duration of smoking cessation required to observe its preventive effect on ARHL in diabetic patients. Prospective observational studies may be needed to clarify details about the prevention of ARHL. Further, the PSM analysis has an innate limitation; in an ideal PSM, it is hypothesized that all possible covariates are included. In this study, 10 validated risk factors from previously published studies were included. However, there may be additional latent covariates affecting the development of ARHL (i.e., genetic polymorphisms) that were not considered in our analyses. Future studies should reflect novel discovered risk factors for ARHL. Additionally, the causal relationship between ARHL and several risk factors could not be clearly elucidated in this study. This is because the KNHANES is a cross-sectional survey, which may have led to a reverse causality bias in this study. However, considering the pathophysiologic mechanism of the medical risk factors associated with ARHL, it is unlikely that ARHL may have increased the risk of medical diseases such as HTN, diabetes, and dyslipidemia. Further, the reduced OR in diabetic ex-smokers compared to current diabetic smokers seems to imply that the cessation of smoking can ameliorate the risk of ARHL; however, this result should not be overestimated since a cross-sectional study cannot fully elucidate the direction of causality. Therefore, further prospective studies may be required in the future to verify the ameliorating effects of smoking cessation. Lastly, the World Health Organization recently defined ARHL as a hearing threshold of > 40 dB HL in 0.5, 1, 2, and 4 kHz frequencies. Although the cut-off threshold of 30 dB HL used in this study has been applied in several publications, the results, especially the OR values, may slightly vary if the cut-off threshold is set at 40 dB HL.

In conclusion, several environmental and medical factors may affect the risk of ARHL. Among them, noise exposure, male sex, and diabetes seem to be important factors, based on both the logistic regression and PSM analyses. In addition to the individual effect of each risk factor, a combinatorial effect of these factors should be considered when attempting to prevent ARHL. As the most modifiable factor, cessation of smoking should be strongly prescribed in high-risk subjects, such as diabetic patients.

## Methods

### Participants

A total of 12,570 individuals (all Korean) included in the 2010 − 2013 KNHANES^18^ participated in this study. The inclusion criteria were normal findings on otoscopic examination, presence of pure-tone audiometry data, and an age of 40–79 years. We excluded individuals who showed a difference of > 30 dB HL in the bilateral pure-tone threshold since ARHL is typically associated with symmetric hearing impairment^4,44^. Individuals with missing values regarding any candidate risk factor [age, sex, HTN, diabetes, dyslipidemia, stroke, CVD, occupational noise exposure, smoking history, and body mass index (BMI)] were also excluded (see Supplementary Figure S1 online).

All study participants included in the 2010 − 2013 KNHANES agreed to participate in the survey and informed consent was obtained for all participants or their legal guardians. The 2010 − 2013 KNHANES was approved by the Institutional Review Board of the Korea Centers for Disease Control (IRB of KCDC). The approval number for the 2010 survey is 2010–02CON-21-C, for the 2011 survey is 2011-02CON-06-C, for the 2012 survey is 2012-01EXP-01-2C, and for the 2013 survey is 2013-07CON-03-4C. KNHANES was performed in accordance with relevant guidelines and regulations provided by the IRB of KCDC.

### Korea National Health and Nutrition Examination Survey

The KNHANES is a cross-sectional survey that has been conducted annually in South Korea since 1998. The fifth KNHANES was conducted from 2010 to 2012, and the sixth KNHANES was conducted from 2013 to 2015. The sample design of the KNHANES was extracted using a two-stage stratified cluster (region and household) sampling. The rate of participation in the fifth and sixth KNHANES was 80.8% and 78.3%, involving 25,533 and 22,948 people, respectively. The KNHANES consisted of three categories: the medical survey, the nutrition survey, and the health examination. Specifically, we only collected datasets that included both noise exposure history and a pure-tone audiogram. Although pure-tone audiometry was conducted from 2008 to 2013, the data from the fourth KNHANES (2007–2009) was not included in this study as it did not include a noise exposure history survey. Since noise exposure history-taking and the pure-tone audiometry test were conducted only from 2010 to 2013, the data from the fifth KNHANES (2010–2012) and the sixth KNHANES (for 2013) were merged vertically to increase the statistical validity. Medical history and smoking information were obtained from the medical survey. BMI and air-conduction pure-tone threshold data were obtained from the health examination.

### Hearing assessment

The subjects underwent pure-tone audiometry in an ordinary soundproof booth that was installed in the vehicle. The test included pure-tone air-conduction threshold measurements at 0.5, 1, 2, 3, 4, and 6 kHz. Individuals with an average pure-tone threshold of ≥ 30 dB HL in the “better” ear at 0.5, 1, 2, and 3 kHz (according to the guidelines of the American Academy of Otolaryngology-Head and Neck surgery foundation^45^) were included in the ARHL group^46,47^. An otorhinolaryngologist examined and reported the tympanic membrane findings in each subject. A well-visualized tympanic membrane without pathologic findings such as retraction, perforation, or middle ear effusion and no cerumen impaction in the middle ear and external auditory canal were defined as normal findings.

### Risk factors

We selected 10 candidate risk factors: age, male sex, HTN, diabetes, dyslipidemia, stroke, CVD, occupational noise exposure, smoking, and obesity. Physician-diagnosed diseases (HTN, diabetes, dyslipidemia, stroke, and CVD) were identified through a self-reported survey. Smoking history was categorized into three groups: never-smoked, ex-smoker, and current smoker. The ex-smoker category indicated that a subject had smoked daily during their lifetime but was not smoking at the time of the survey. BMI was converted into a dichotomous variable termed “obesity”; a BMI > 25 kg/m^2^ was considered obese^48^. Occupational noise exposure history was recorded via a survey question asking if participants had worked in a noisy environment (difficulty in communication with moderate loudness [55 dB HL] between workers) for > 3 months. Information on age and sex was obtained from residence registration data.

### Statistical analysis

The 10 candidate risk factors were treated as interacting covariates, and logistic regression and PSM analyses were performed with the same sample and covariates. In order to classify the most associated determinants, the logistic regression analysis was performed using the stepwise backward selection (cut-off *p* value = 0.05) method with the Hosmer‒Lemeshow test (valid if *p* value > 0.05). The accuracy of classification and Nagelkerke’s R^2^ were calculated to evaluate the explanatory power. ARHL was included as a dependent variable, and the 10 risk factors were included as independent variables in the regression model.

Propensity scores were calculated using logistic regression analysis, which included the target risk factor as a dependent variable and other risk factors as independent variables. We performed 1:1 matching between individuals with the same propensity score to obtain two exact propensity score-matched groups. From the matched pairs, ORs and 95% confidence intervals (CIs) were calculated from a 2 × 2 table, including ARHL (presence or absence) and the target risk factor (presence or absence). We defined two groups to evaluate the ORs of the combined risk factors: one was the combined risk group with both risk factors, and the other was a control group with one or no risk factors. After classification, PSM and OR calculation was performed in the aforementioned manner.

The McNemar’s test was performed to evaluate the statistical significance of the difference in ARHL risk factors between the propensity score-matched groups. The pure-tone threshold of each frequency was compared using two-way analysis of variance with Sidak’s post-hoc. A *p* value < 0.05 was considered statistically significant. The statistical analyses were conducted using Statistical Package for the Social Sciences version 25.0 (IBM; Armonk, NY).

## Supplementary information

Supplementary Information.

## Data Availability

The data are available from the corresponding author upon reasonable request.
