# Supplementary material for: Synergistic effect of smoking on age-related hearing loss in patients with diabetes
Source: Sci Rep. 2020 Nov 3;10:18893. doi: 10.1038/s41598-020-75880-2 (PMC7641162; doi:10.1038/s41598-020-75880-2)
Supplement: Supplementary file 1 — Supplementary Information. [file 41598_2020_75880_MOESM1_ESM.docx]

**Supplementary Material**

**Synergistic effect of smoking on age-related hearing loss in patients with diabetes**

Seong Hoon Bae^1^, Sang Hyun Kwak^2^, Jae Young Choi^1^, Jinsei Jung^1^*

**File Contents:**

**Supplementary Figure S1.** Flow chart of enrollment. The criteria and number of included/excluded individuals are presented in each box.

**Supplementary Table S1.** Demographic data of the included individuals.

**Supplementary Table S2.** Propensity score-matched groups (current smokers vs. individuals who have never smoked).

**Supplementary Figure S1.** Flow chart of enrollment. The inclusion/exclusion criteria and the number of included/excluded individuals are presented in each box. KNHANES, Korea National Health and Nutrition Examination Survey.

**
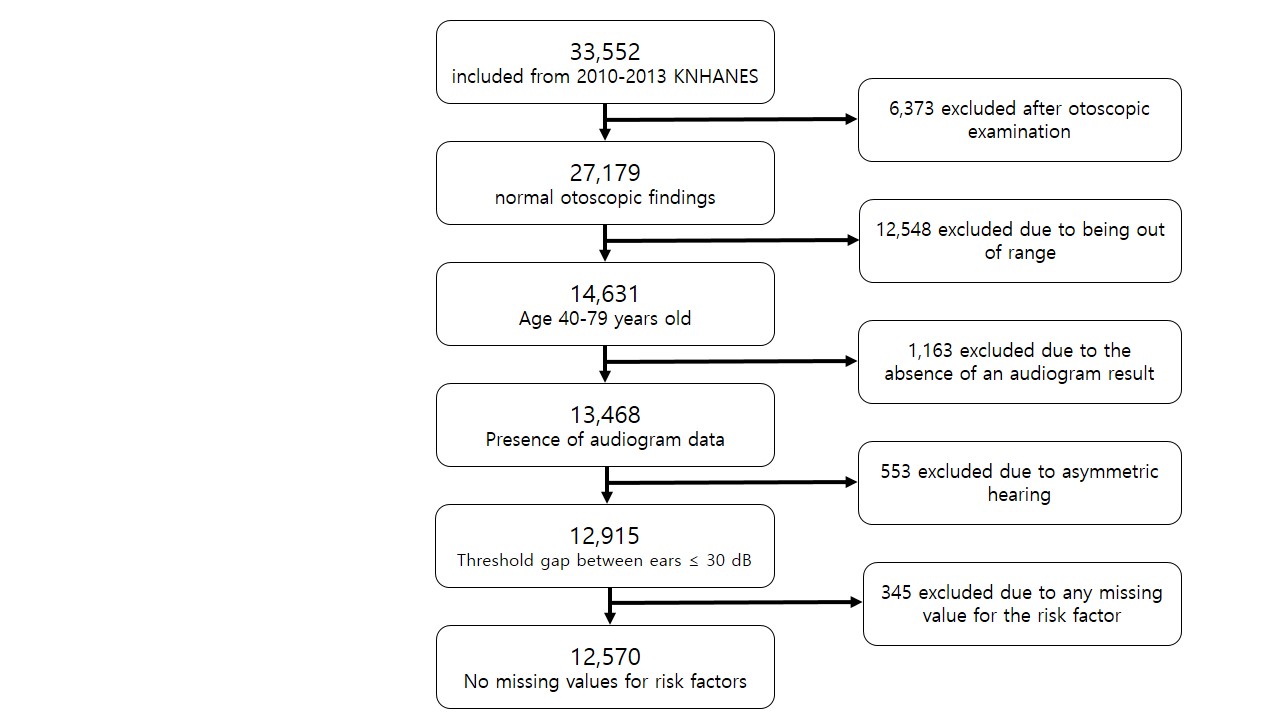
**

**Supplementary Table S1.** Demographic data of the included individuals

| Characteristics | N (%) |  |
| --- | --- | --- |
| Number of included individuals | 12,570 | |
| Age-related hearing loss | 2,002 (15.9%) | |
| Age (mean ± standard deviation), years | 57.21 ± 10.86 | |
| Male sex | 5,482 (43.6%) | |
| Hypertension | 3,720 (29.6%) | |
| Diabetes mellitus | 1,408 (11.2%) | |
| Dyslipidemia | 1,983 (15.8%) | |
| Stroke | 297 (2.4%) | |
| Cardiovascular disease | 441 (3.5%) | |
| Occupational noise exposure | 1,755 (14.0%) | |
| Obesity | 4,447 (35.4%) | |
| Current-/Ex-Smoker/Never-Smoked | 2,296 (18.3%) / 2,859 (22.7%) / 7,414 (59.0%) | |

**Supplementary Table S2.** Propensity score-matched groups (current smokers vs. individuals who have never smoked)

| Variables | Current smoker | Never smoked | *p*-value |
| --- | --- | --- | --- |
| Number | 950 | 950 | N/A |
| Age (mean ± standard deviation), years | 55.56 ± 11.09 | 55.56 ± 11.09 | 1.0 |
| Male sex | 672 | 672 | 1.0 |
| Hypertension | 198 | 198 | 1.0 |
| Dyslipidemia | 73 | 73 | 1.0 |
| Stroke | 6 | 6 | 1.0 |
| Cardiovascular disease | 0 | 0 | 1.0 |
| Diabetes mellitus | 70 | 70 | 1.0 |
| Smoking (current/ex/never) | 950/0/0 | 0/0/950 | N/A |
| Obesity | 288 | 288 | 1.0 |
| Occupational noise exposure | 122 | 122 | 1.0 |
| Age-related hearing loss | 126 | 149 | 0.151 |

N/A, Not applicable
